# Enhancing Pragmatic Language skills for Young children with Social communication difficulties (E-PLAYS-2) trial: study protocol for a cluster-randomised controlled trial evaluating a computerised intervention to promote communicative development and collaborative skills in young children

**DOI:** 10.1186/s40359-024-01749-y

**Published:** 2024-05-13

**Authors:** Suzanne Murphy, Kerry Bell, Erica Jane Cook, Sarah Crafter, Rosemary Davidson, Caroline Fairhurst, Kate Hicks, Victoria Joffe, David Messer, Lyn Robinson-Smith, Luke Strachan, David Torgerson, Charlie Welch

**Affiliations:** 1 University of Bedfordshire, University Square, Luton, Bedfordshire LU1 3JU UK; 2 York Trials Unit, University of York, Heslington, York YO10 5DD UK; 3 Open University, Walton Hall, Kents Hill, Milton Keynes, Milton, MK7 6AA UK; 4 University of Essex, Wivenhoe Park, Colchester, CO4 3SQ UK

**Keywords:** Social communication, Pragmatic language, Randomised controlled trial, Feasibility study, Young children, Peer collaboration, Communication impairment, Computer game

## Abstract

**Background:**

A number of children experience difficulties with social communication and this has long-term deleterious effects on their mental health, social development and education. The E-PLAYS-2 study will test an intervention (‘E-PLAYS’) aimed at supporting such children. E-PLAYS uses a dyadic computer game to develop collaborative and communication skills. Preliminary studies by the authors show that E-PLAYS can produce improvements in children with social communication difficulties on communication test scores and observed collaborative behaviours. The study described here is a definitive trial to test the effectiveness and cost-effectiveness of E-PLAYS delivered by teaching assistants in schools.

**Methods:**

The aim of the E-PLAYS-2 trial is to establish the effectiveness and cost-effectiveness of care as usual plus the E-PLAYS programme, delivered in primary schools, compared to care as usual. Cluster-randomisation will take place at school level to avoid contamination. The E-PLAYS intervention will be delivered by schools’ teaching assistants. Teachers will select suitable children (ages 5–7 years old) from their schools using guidelines provided by the research team. Assessments will include blinded language measures and observations (conducted by the research team), non-blinded teacher-reported measures of peer relations and classroom behaviour and parent-reported use of resources and quality of life. A process evaluation will also include interviews with parents, children and teaching assistants, observations of intervention delivery and a survey of care as usual.

The primary analysis will compare pragmatic language scores for children who received the E-PLAYS intervention versus those who did not at 40 weeks post-randomisation. Secondary analyses will assess cost-effectiveness and a mixed methods process evaluation will provide richer data on the delivery of E-PLAYS.

**Discussion:**

The aim of this study is to undertake a final, definitive test of the effectiveness of E-PLAYS when delivered by teaching assistants within schools. The use of technology in game form is a novel approach in an area where there are currently few available interventions. Should E-PLAYS prove to be effective at the end of this trial, we believe it is likely to be welcomed by schools, parents and children.

**Trial registration:**

ISRCTN 17561417, registration date 19th December 2022.

Protocol version: v1.1 19th June 2023.

## Background

Children who have difficulties with social communication (also known as pragmatic language ability) experience problems with using language for social purposes. Whilst their knowledge of grammar and vocabulary may be adequate or even advanced, they struggle with communicative tasks such as appropriate use of greetings, conversational turn-taking, understanding non-literal language such as jokes, irony or sarcasm, social conventions such as politeness, taking the perspective of their listener and responding with relevant information [1].

'Social communication difficulties' (SCDs) or 'pragmatic language impairments' represent a continuously distributed trait in the population. This trait includes individuals at the extreme end who are diagnosed with autistic spectrum disorder and/or severe language disorders but also a much larger group who show milder, but still detrimental, communication difficulties [2].

Children with SCDs are commonly rejected and victimised by peers [3, 4] and can be severely disruptive [5–7]. In groups, they fail to contribute appropriately, and are often ignored or dominated by peers [8, 9]. Children with pragmatic language problems experience lower quality of life; in adulthood these individuals experience more mental health problems, lower academic achievement and make fewer friends [10]. Health economic evaluations have also been called for as healthcare costs have been shown to be 36% higher for children with language disorders at 4–5 years old [11].

These communication difficulties frequently cause troubled interactions with family, peers, teachers and the criminal justice system [12, 13]. For primary school children of low socio-economic status, pragmatic language skills appear to be especially important [14].

Children with language difficulties in the UK are served by NHS Speech and Language therapists and/or by schools’ own speech and language services and schools’ other provisions. However, services are stretched, particularly since the pandemic. Furthermore, schools and speech and language therapists have few rigorously tested interventions that they can use for SCDs. The most recently available surveys of usual care reported a ‘proliferation of locally-developed programmes based on clinical experience’ due to a lack of ‘strongly evidence-based programmes’ [15, 16]. These findings were borne out by interviews with schools and speech and language therapists in our earlier work [17]. Activities typically include exercises on turn-taking, topic management, and conversational skills, sometimes with role-play or modelling. There is little evidence concerning the efficacy of these constituent activities [16]. Whilst the use of technology and gaming has been highlighted as a positive tool for facilitating communication and collaboration in children with social communication difficulties [18, 19], its use is generally viewed as emerging rather than established [20].

E-PLAYS (Enhancing Pragmatic Language skills for Young children with Social communication difficulties) is a computer-based intervention that has been developed and piloted by our team.

Collaborative and team-building skills are recognised as vital to future adult employment and participation in society [21]. However, some of the most challenging contexts for children with social communication difficulties are precisely those requiring collaboration, such as joint problem-solving or creative free play [8, 9, 22, 23]. E-PLAYS aims to facilitate and enhance children’s interactions by providing socio-cognitive scaffolding within a fun, cooperative computer game.

E-PLAYS supports communication based around naturalistic play with a peer and aims to embed learning in relevant contexts, thus promoting the generalisation of these social skills.

An earlier version of E-PLAYS (known as the Maze Game [9, 23]) was tested on 32 children. Children receiving the intervention showed significant improvement by comparison to a control group on pragmatic language test scores. A recent feasibility study of E-PLAYS [17] with 50 children showed good response and completion rates, realistic recruitment and high acceptability by children and schools. These studies laid the groundwork for the present study which will conduct a randomised controlled trial of E-PLAYS seeking to establish its clinical- and cost-effectiveness.

## Design and methods

### Aim

The aim of the E-PLAYS-2 trial is to establish the effectiveness and cost-effectiveness of care as usual plus the E-PLAYS programme, which is designed to improve pragmatic language skills in children with social communication difficulties, delivered in primary schools, compared to care as usual.

### Trial design

The E-PLAYS-2 trial is a multi-centre, two-arm, cluster-randomised controlled trial with an internal pilot.

### Setting

The trial will take place in state-funded mainstream primary schools and state-funded special primary schools in Bedfordshire, Hertfordshire and North London. Following slightly lower than anticipated recruitment during the internal pilot phase, primary schools in Buckinghamshire will also be recruited for the main trial.

### Participants

Both the school and the children’s parents/carers must agree to take part before either may be included. Eligibility to take part will be ascertained using the following criteria.

### School eligibility

#### Inclusion criteria:

A state-funded infant or primary school or special needs school based in the target recruitment areas;

#### Exclusion criteria:

- Independent, fee-paying schools;
- Schools participating in other language and communication research/trials aimed at pupils in Year 1 and Year 2 (aged 5–7 years);
- Schools who have previously used E-PLAYS;

### Child participants

Teachers will use the Social Communication Behaviour Checklist [24] which comprises a short 5-item questionnaire to confirm or reject their selection for ‘Focal’ children. Similarly, teachers will use the Social Communication Behaviour Checklist to confirm the selected ‘Partner’ children do *not* meet the criteria for social communication difficulties (see ‘Intervention’ section for definitions of Focal and Partner children). Child recruitment will take place prior to school randomisation.

### Child eligibility (Focal children)

Focal child eligibility criteria are as follows:

- Children aged 5–7 years old;
- Children who meet the criteria for social communication difficulties as determined by the Social Communication Behaviour Checklist [24];
- Children whose parents/carers give consent for them to take part in the E-PLAYS-2 trial;
- Children who have not used E-PLAYS before;
- Children whose parents/carers are willing to complete relevant questionnaires;
- Children who complete the key trial baseline assessments (assuming all other eligibility criteria are met);

Baseline and outcome data will be sought for all Focal children (subject to potential withdrawals from some or all aspects of follow-up data collection by participating schools or parents).

### Child eligibility (Partner children)

Partner child eligibility criteria are as follows:

- Children aged 5–7 years old;
- Children who do *not* meet the criteria for social communication difficulties as determined by the Social Communication Behaviour Checklist [24];
- Children whose parent/carers give consent for them to take part in the E-PLAYS-2 trial.

Not all Partner children will complete assessments. We will randomly select one Partner child from each school to complete the Test of Pragmatic Skills (TPS) at baseline and follow-up assessments (see details of the TPS below). This will allow for a comparison of the outcomes in this subsample of typically-developing children between intervention (where the child will partner a participating child in E-PLAYS-2) and control schools (care as usual). Parents/carers of the Partner children will be asked to consent to the Partner child completing the TPS (although only one randomly selected participant in each school will complete these assessments as stated above).

### Intervention

The E-PLAYS programme is designed for children with SCDs aged 5–7 years old (referred to hereafter as ‘Focal’ children). Using a computer game, E-PLAYS guides the Focal child through real-life conversational exchanges with a specific focus on (a) requesting optimally useful information (b) giving helpful directions and (c) asking for clarification. Each Focal child is matched with a ‘Partner’; a typically-developing child from the same year group.

Each E-PLAYS session uses the computer game which is designed for two players using interlinked laptops. There are ten weekly sessions of 30 min each; schools’ teaching assistants are trained to deliver and supervise all sessions. Five sessions are with the Focal child and the teaching assistant only, five are with the Focal and Partner child together supervised by the teaching assistant. Sessions with the classmate (Partner child) give the child an opportunity to practice newly-acquired skills and also to learn collaboration skills through joint problem-solving with a peer. E-PLAYS is web-based, enabling us to distribute E-PLAYS directly to schools. Teaching assistants will self-train using a comprehensive manual with online support. The E-PLAYS software automatically records the number of sessions along with date accessed and sends this data directly to the research team.

### Recruitment

#### School recruitment

Recruitment strategies include directly emailing schools based in the target recruitment areas, use of social media channels and working with contacts in relevant local authorities by providing them with recruitment materials to distribute at a local level. During initial contact, schools will be provided with an information sheet about the trial. Where schools express an interest in participating, a member of the research team will arrange a convenient time discuss the trial with an appropriate staff member (e.g., a Head Teacher or a Special Educational Needs Co-ordinator (SENCO)) in greater detail. Schools wishing to proceed will be required to sign a memorandum of understanding (MoU) agreeing to the expectations of the trial, and a Data Sharing Agreement (DSA) between the school and the research team.

#### School retention and withdrawal

Schools will receive £350 as a thank you for taking part in the trial. This will be paid in instalments by the University of Bedfordshire after key milestones have been reached (such as completion of mid-trial surveys).

Where a school indicates that they wish to withdraw from the study this will result in the full withdrawal of all participants and staff at this school. No further data will be collected. The school will inform the parents/carers that they have withdrawn.

#### Child recruitment

Once teachers have identified the children eligible to take part in the trial, the teacher will distribute paper information sheets and consent forms to their parents/carers. Translated versions will be offered for parents with English as an additional language (EAL). The participant information sheets will be supplied to schools by the research team, along with a simplified illustrated information sheet for children to read together with their parents/carers. The information sheets and consent forms will be tailored to Focal and Partner children. Schools will be asked to send a reminder to parents/carers if no response is received approximately two weeks after receipt of the original invitation pack. Completed consent forms are to be returned to the school for collection by the research team.

#### Child consent procedure

All parents will be given the option to speak to a member of the research team or to contact the Chief Investigator in the event of additional questions. Consent to enter the study will be sought from each participant only after a full explanation has been given, an information leaflet offered and time allowed for consideration.

Participation in the study will be entirely voluntary and written informed consent from parents/carers will be obtained before child baseline data is collected and randomisation is conducted. On the consent form, parents/carers will be requested to consent for their child’s school to provide data including child’s name, date of birth, gender, home postcode, ethnicity, religion/belief, English as an additional language (EAL), education, health and care plan (EHCP) status, help received from a Speech & Language Therapist and/or an Educational Psychologist and receipt of Pupil premium and/or free school meals (FSM, a proxy for deprivation) for the purposes of sample description and potentially for use as covariates in analyses. The consent form for Focal children will also request parent/carers to provide their educational qualifications, employment status, ethnicity and consent/commitment to complete the EQ-5D-Y (proxy version 1), CHU-9D, and resource use data questionnaires at specified time-points. Parents/carers will return completed consent forms to the school.

#### Child and parent/carer retention and withdrawal

At the end of the trial and following completion of all questionnaires, parents/carers of Focal children will receive a £15 voucher to offset any incidental expenses and in recognition of their participation.

All participants are free to withdraw at any time from either the intervention or follow-up data collection without giving reasons and without prejudicing further care. The Chief Investigator will preserve the confidentiality of participants taking part in the study and is registered under the Data Protection Act. If a child does not appear to want to take part at the time the E-PLAYS intervention is being delivered and/or assessments are taking place, their wishes will be respected. Where a parent/carer wishes to withdraw from the study, it will be clarified as to whether they wish their child to withdraw from the intervention or if they themselves wish to withdraw (i.e., stop completing outcome measures). Where withdrawal is only for the participating parent/carer, the child may continue to take part in all other aspects of the trial and follow-up data will continue to be collected when possible. If a Partner child withdraws, another child from the school will be recruited as a replacement for the purposes of intervention delivery.

#### Teaching assistant recruitment

All teaching assistants will be asked to provide information at baseline on their work training and experience. School staff will also be asked to complete a survey exploring usual care for children with social communication difficulties. A subset of teaching assistants will be asked to participate in interviews, observations and focus groups; for these a separate information sheet and consent form will be provided by the research team.

#### Teaching assistant retention and withdrawal

Where withdrawal is only for the teaching assistant, we will ask schools to replace them for the intervention period. Where a teaching assistant cannot be replaced, the study team will discuss the implications of this with affected participant(s) to establish if they wish to continue providing outcome data.

### Randomisation

The trial will be cluster-randomised to prevent within-school contamination. Schools will be allocated to intervention or control group using minimisation based on geographical location and proportion of children with free school meals (a proxy for deprivation) by a trial statistician at York Trials Unit using dedicated software (MinimPY [25]). Randomisation will occur following baseline data collection. Schools will be informed of their allocation via email. Schools will be advised to tell the parents/carers of participating children the schools random allocation.

Children in schools allocated to the intervention group will receive E-PLAYS plus whatever constitutes care as usual in their school. Participating children in schools allocated to the control group will receive whatever constitutes care as usual in their school. ‘Care as usual’ is defined as the existing support routinely provided for a child with social communication difficulties from educational and health services.

### Outcome measures

Outcome data will be provided by three different kinds of observers: blinded research assistants, parents/carers and teachers.

#### Blinding of outcome data collection

Research assistants will be blind to group allocations when collecting the quantitative outcome measures listed below. They will have received relevant training from the research team. All research assistants will have an enhanced Disclosure and Barring Service (DBS) check and undergo relevant safeguarding and data protection training. When a research assistant visits a school to administer the assessments, teachers and teaching assistants at the school will be reminded on every visit not to reveal allocation to the research assistants. Any instances of unblinding during the assessments will be recorded (using a bespoke unblinding form which will include information on who was unblinded, the source of unblinding, and the reason for unblinding) and the unblinded research assistant will be replaced with another research assistant who is blind. Research assistants will also collect qualitative data from schools; for this data, they will not be blinded. Hence, each school will be allocated both a blinded and unblinded research assistant.

Teachers and parents/carers will be requested to complete outcome measures for Focal children. Whilst blinded during the completion of these outcome measures at baseline, due to the nature of the intervention, it is not possible for them to be blinded at 15–20- or 35–40-weeks post-tests.

#### Primary outcome

The primary outcome is the Focal children’s pragmatic language ability measured using the validated Test of Pragmatic Skills (TPS [26]). This assessment will be administered by a blinded research assistant at baseline, and at 15–20- and 35–40-weeks post-randomisation. The measurement at 35–40 weeks will serve as the primary endpoint for the trial, with the 15–20-week measurement being a secondary outcome.

#### Secondary outcomes

The following secondary outcome measures will also be administered to Focal children by a blinded research assistant at baseline, 15–20 weeks, and 35–40 weeks post-randomisation.

- Clinical Evaluation of Language Fundamentals-5 (CELF-5 [27])—Recalling Sentences and Following Directions subscales.
- Expression, Reception and Recall of Narrative Instrument (ERRNI [24]) assesses the ability to relate, comprehend and remember information after a short delay.
- Droodles Tasks and Communication Test [28, 29].

The Droodles Task and Communication Test are a series of tasks and puzzles testing children’s ability to evaluate the effects of ambiguous versus informative communications, a key skill targeted by E-PLAYS. The tests are embedded in play sessions with dolls and puppets and have previously been used for this age group.

The battery of assessments above will take approximately 50 min to administer per child at each data collection time-point. The children’s tests are mostly tasks presented as fun games to play and therefore not onerous for the children. These tests can be divided into two or more sessions as the children are very young and may tire.

The following secondary outcome measures (relating to health-related quality of life) will be completed by Focal children’s parents/carers at baseline, 15–20 weeks and 35–40 weeks post-randomisation:

- Child Health Utility (CHU-9D), paediatric generic preference-based measure of quality of life. The CHU-9D includes specific dimensions on school and joining in with activities [30, 31].
- EQ-5D-Y (proxy version 1). This is a widely used standardised generic measure of health-related quality of life for younger children [32].
- Resource use data: A bespoke questionnaire (developed for the E-PLAYS feasibility study [17]) will collect resource use data about healthcare, voluntary organisations and educational resources.

We anticipate that it will take parents/carers approximately 30 min to complete the questionnaires at each data collection time-point.

The following secondary outcome measures will be completed by the Focal children’s teacher at baseline, 15–20 weeks and 35–40 weeks post-randomisation; these measures are completed by the teachers without the child present:

- Children’s Communication Checklist-2 (CCC-2[33]). CCC-2 is a standardised questionnaire of children’s communication impairment.
- The Strengths and Difficulties Questionnaire (SDQ [34]). The SDQ is widely used as a mental health indicator with subscales assessing behavioural, emotional and peer problems.

We anticipate the questionnaires listed above will take teachers no longer than ten minutes per child to complete at each data collection time-point.

The following secondary outcome measures will be administered to a randomly selected subgroup of 88 Partner children (1 per school) in school by a research assistant at baseline, 15–20 weeks and 35- 40 weeks post-randomisation:

Partner children’s pragmatic language ability measured using the validated TPS [26].

### Sample size calculations

#### Original

We will recruit single- and multi-form entry schools. Pupils will be recruited from Years 1 and 2; assuming an average of two classes per year, based on our feasibility study [17] we expect to identify a mean of ten eligible Focal children per school, of which six will consent and be recruited. The intervention will be delivered to the participating children by teaching assistants and we expect an average of 1.5 teaching assistants per class.

In multi-form entry schools, we will have clustering of classes within year groups, but in one-form entry schools the levels of class and year will be equivalent. We consider that in multi-form entry schools the difference in clustering between class and year will be negligible so we shall ignore the level of class. Therefore, this cluster randomised trial assumes a three-level structure in that pupils (level 1) are nested within year group (level 2) nested within schools (level 3). Randomisation will take place at school-level. The year groups participating in this trial are consecutive (Years 1 and 2) so the difference between them will be minimal and the cluster effect of school will likely dominate the effect of class; therefore, we have not explicitly accounted for clustering at the class level in this sample size calculation. The largest influence within schools is likely to be between teaching assistants since these will be the ones delivering the intervention to the children; however, in most schools we expect that the ratio of teaching assistants to participating children will be approximately 1:1 so this level of clustering is eliminated. In the feasibility trial [17], the school-level ICC was small (< 0.01); here we have assumed a conservative ICC of 0.05 at the school-level to account for all levels of potential clustering.

In our feasibility trial the standard deviation (SD) of the primary outcome measure, the TPS [26], at baseline was 7.2 (95% CI 5.4 to 9.7) and the observed correlations between the TPS score at baseline and the scores at weeks 15–20 and 35–40, respectively, were 0.84 (95% CI 0.71 to 0.91) and 0.79 (95% CI 0.63 to 0.89). In the calculation for this trial we assume: a SD of 7, an ICC of 0.05 at the school-level, a mean cluster size of six (Focal children per school, at randomisation), 20% pupil level attrition at follow-up and a more conservative pre-post correlation of 0.6. To detect a difference in TPS score of 2 points (a third of a year’s progress based on the standardisation sample given in the TPS manual), with 90% power and a two-sided alpha of 5%, we would require 84 schools (504 focal children).

We will undertake an exploratory analysis to assess the potential impact of the intervention on Partner children’s (i.e., those who do not have social communication difficulties) pragmatic language skills. We will randomly select one Partner child from each school to complete the TPS [26] at baseline, at 15–20 weeks post-randomisation and at 35–40 weeks post-randomisation with a blinded, independent research assistant. This will allow for a comparison of the outcomes in these typically-developing children between intervention (where the child will partner a participating Focal child in E-PLAYS) and control schools (care as usual).

Since this is an exploratory analysis, we have planned the sample size of one typically-developing child from each school for logistical reasons. Collecting the TPS [26] from only one extra child per school will not substantially increase the time or burden to complete outcome measures. Assuming a SD of 7, a pre- post-test correlation of 0.6 and 20% attrition, a sample size of 84 children (one per school) will give 80% power to detect a difference of 3.9 points in the TPS [26]. We shall compare TPS [26] scores of the typically developing children.

#### Revised

For the 20 school clusters recruited as part of the internal pilot phase, the observed mean cluster size (at randomisation) was 4.55 participants per cluster, around 25% less than the anticipated six participants per cluster detailed in the previous section.

Following discussion with the funder, the total target number of school clusters was changed to 88. Assuming a mean cluster size (at randomisation) of 4.55 and keeping all assumptions the same as previously (e.g.$\delta$ = 2, SD = 7, pre-post correlation = 0.6, school level intra-cluster correlation of 0.05 and 20% participant level attrition), 44 clusters per group would provide approximately 85.2% power for a two sided test of ${H}_{0}:\delta =0$ (where $\delta$ is the difference in expected TPS score at 35–40 weeks).

As per the original proposal we will randomly select one Partner child per school to complete the primary outcome (TPS [26],) at baseline and 15–20- and 35–40-weeks post-randomisation. Under the same assumptions as before, a sample size of 88 children (one per school) will give 80% power to detect a difference of 3.4 points in the TPS [26].

### Statistical analysis plan

Statistical analysis will primarily be conducted in Stata/MP v18 [35] or later, unless specified otherwise. All analyses will be conducted just once at the end of the trial follow-up period, according to precise specifications detailed in a Statistical Analysis Plan (SAP) that will be approved by the TMG and TSC prior to the end of follow-up. Any departures from the analysis plan will be reported and justified in the final trial reports and other relevant published articles.

The flow of clusters and participants through the study will be presented according to CONSORT guidance for cluster RCTs. Continuous characteristics will be summarised in terms of the available sample size, arithmetic mean, standard deviation, median, interquartile range, minimum and maximum. Categorical characteristics will be summarised in terms of frequencies and percentages.

For all between group comparisons, clusters and participants (both Focal and Partner) will be analysed as part of the groups to which they were randomised, regardless of subsequent engagement with the allocated treatment. All analyses estimating between group contrasts will include all participants with data available for the relevant outcome (unless explicitly stated otherwise in the SAP). Point estimates of contrasts between randomised groups will be reported together with appropriate 95% confidence intervals. Point and interval estimates will be reported on the scale of the original measurements (as well as the scale used for the analysis should these differ). P-values for statistical tests will be two-sided unless specified otherwise in the SAP.

### Baseline data participants

Baseline data for the participating focal children will be summarised descriptively by randomised group and overall, according to the principles outlined above. Two sets of tables will be reported: one set including all randomised Focal children and another including just the subset of Focal children included in the primary analysis model. Baseline data for the participating Partner children will be summarised similarly in a separate set of tables. No formal comparison of baseline data between randomised groups will be undertaken (for either Focal or Partner children).

### Primary outcome analysis (focal children)

The planned primary analysis model will include all available post-randomisation TPS [26] scores as outcomes, modelling these measurements using a linear mixed effect model. This model will include fixed effects for treatment group, time point, and their interaction, and will also condition on fixed effects for baseline TPS mean composite score, year group, child FSM status, geographical location of school, and school level random intercepts. Correlation between repeated measurements within participants will be modelled using an unstructured covariance matrix for the model residuals. Precise details of the terms included in the model will be provided in the SAP (including plans for dealing with any incomplete baseline covariate data). If the fit of the planned primary analysis model is reasonable (see below), then the fitted model will be used to estimate differences (Intervention – Control) in expected TPS scores at both post-randomisation time points, together with 95% confidence intervals and p-values for tests of H_0_: $\delta$ = 0 (where $\delta$ is the difference in expected score at the relevant time point).

The appropriateness of key model assumptions will be checked using diagnostic plots based on the standardised residuals from the fitted model. If these plots (or indeed other extra-data considerations) suggest the observed data show important departures from the assumptions of the planned analysis, then we will undertake semi-parametric analyses of the scores at each post-randomisation time point in isolation. This will be accomplished using cumulative probability models based on ordinal regression [36]. The ordinal regression models will include fixed effects for treatment group, baseline TPS [26] mean composite score, year group and child FSM status, and a random intercept for school.

### Sensitivity analyses (Focal children)

Several additional planned analyses of the primary outcome will be undertaken to investigate the sensitivity of the results of the primary analysis to departures from the key statistical assumptions that underpin this analysis. In particular, we will investigate the impact that various alternative adjustment sets have on the results of the primary analysis, investigate the potential impacts of departures from the planned schedule of assessments, and undertake analyses of the primary outcome under different assumptions about any missing primary outcome data.

### Principal stratum analyses (Focal children)

Participants allocated to the control group will not have access to the E-PLAYS intervention. Participants allocated to the intervention group will be offered the E-PLAYS intervention, but may not receive any sessions at all, or may receive only a proportion of the planned ten sessions (or potentially none of them).

We will estimate two different CACE estimands under different definitions of compliance: (1) The difference in expected TPS mean composite score at 35–40 weeks among participants that would complete at least one session of the E-PLAYS programme if they were randomised to E-PLAYS; (2) The difference in expected TPS mean composite score at 35–40 weeks among participants that would complete at least seven E-PLAYS sessions (70% of the programme) if they were randomised to E-PLAYS.

We will use instrumental variable estimators to estimate both of these principal stratum estimands. Specifically, we will use random allocation as an instrument for the relevant definitions of treatment receipt in each case, with estimation performed using a generalised two-stage least squares random effects estimator. These analyses will include the same baseline covariates as included in the primary analysis and random intercepts for school cluster. Point estimates of the two principal stratum estimands outlined above will be reported together with two-sided 95% confidence intervals and p-values for tests of H_0_: $\delta$ = 0.

### Secondary outcome analyses (Focal children)

Continuous secondary outcomes will be analysed similarly to the primary outcome, with the baseline TPS score replaced with the baseline score for the relevant outcome. Ordered categorical secondary outcomes will be analysed using appropriate ordinal regression models with similar fixed and random effects as included in the primary analysis.

### Fidelity analysis

E-PLAYS software will record the content, duration and number of intervention sessions each child receives using a unique login ID. This monitoring data will be summarised as part of the process evaluation and used to estimate Complier Average Causal Effect (CACE) estimands (see above).

### Primary outcome analysis (Partner children)

**Fig. 1:**
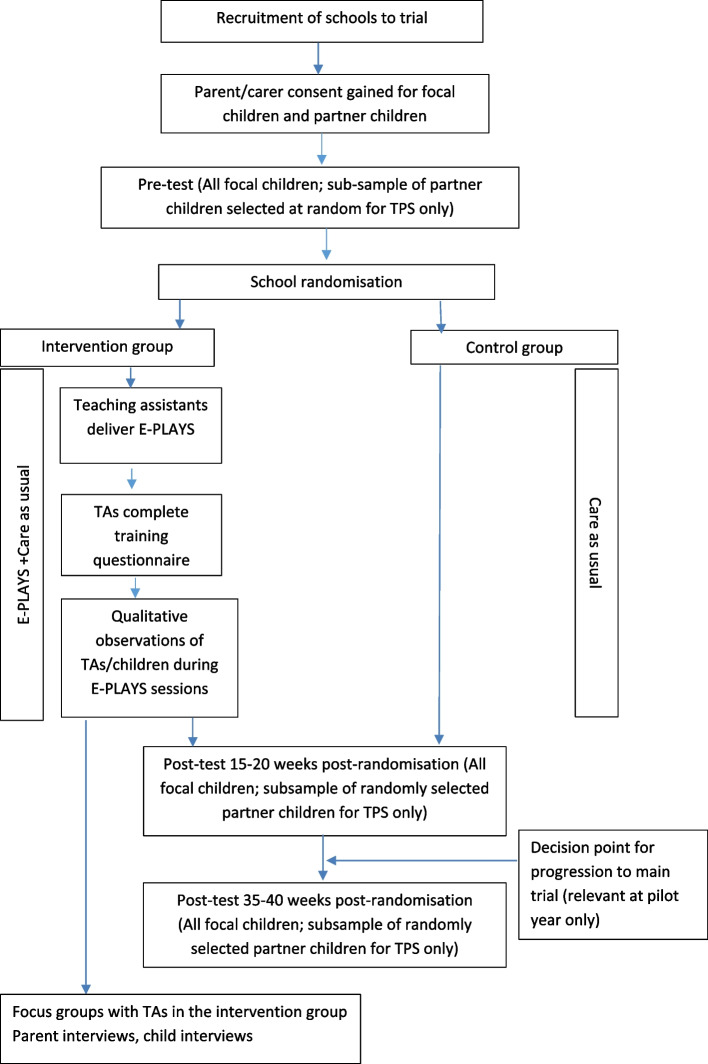
Flow diagram of the E-PLAYS-2 recruitment, randomisation and data collection

The TPS scores for Partner children will be analysed following a similar approach to the primary analysis but will not include random intercepts for school cluster (since there will be no replication at the participant at within cluster level) (Fig. 1).

### Process evaluation

A mixed-methods process evaluation, following MRC recommendations for RCTs [37], will assess E-PLAYS' acceptability and fidelity of implementation, mechanism of impact, and examine contextual influences on implementation and outcomes. This evaluation will use quantitative and qualitative data across the school sample alongside observation, interview and focus group data from four purposively selected case study schools. Research assistants will conduct the interviews, observations and focus groups described below.

### Case study schools

Eight intervention schools (four from the internal trial and a further four from the main trial) will be purposively sampled to act as case studies [38]. Schools will be profiled to include at least the following: one special needs and one mainstream school plus one school with high levels of deprivation and another with a high proportion of children with English as an additional language. These schools will be approached to be case studies before the E-PLAYS intervention is given to them and will continue to be observed throughout intervention delivery. The following assessments will take place with a subgroup of case study participants:

- Structured observations of the children (Focal and Partner) and teaching assistants as they use E-PLAYS, based on an observation schedule developed for the E-PLAYS feasibility study ([17] mid-intervention);
- Focus groups conducted with teaching assistants exploring their experiences of delivering E-PLAYS (end intervention);
- Interviews with the children (Focal and Partner) with a card sorting task and visual analogue scale to give an indication of their liking of E-PLAYS (mid-intervention);
- Structured interviews with parents exploring the extent to which children play computer games at home before and after the intervention and any changes to game-playing (baseline and 40-week follow-up);

### Surveys

In addition, a training questionnaire will be sent to all teaching assistants delivering E-PLAYS to obtain feedback on the training manual and online support. This survey will also include questions on the teaching assistants training and experience. A further survey will be sent to all participating schools based on our findings from the E-PLAYS feasibility study [17], asking about the content of usual care for children with SCD. We will also include 6–8 structured interviews with a subset of teaching assistants to further explore usual care provided. Surveys will be delivered via Qualtrics online survey software, with a paper version available on request. Written consent will be obtained from teaching assistants to participate in focus groups and interviews.

### Process evaluation analyses

Qualitative data will be (with written consent) audio-recorded, transcribed verbatim and managed using NVivo11 software. A six-step reflexive thematic analysis approach [39] will be used to report the experiences, meanings, and reality of participants. Two experienced qualitative researchers will independently code a subsample of transcripts where initial codes will be compared, discussed, and agreed on prior to coding on all other interviews. Codes will be generated both from the topics explored in the interview guides and iteratively from the data to attain both the facilitators and challenges of the intervention. Interim themes will then be discussed, refined, and agreed by two researchers and the research team. Detailed analysis of each theme will be presented with illustrative anonymised quotes used to illustrate themes arising from the data. Individual interview and focus group data will be analysed both separately, followed by a cross-synthesis, to identify and map overarching themes related to experiences of the intervention. Comparative analysis across the case study schools will also be conducted to explore the impact of the intervention and examine experiences across different school contexts.

### Economic evaluation

The costing approach will be undertaken primarily from the perspective of the National Health Service (NHS) but will also consider the perspective of both Social and Education Services. The economic evaluation will assess the cost-effectiveness of E-PLAYS compared with usual care. Individual participant data from the trial will be used to evaluate resource use, costs, health and social outcomes associated with the intervention and will be collected over the follow-up period of the trial.

The primary economic outcome will be the difference in costs and the difference in quality-adjusted life years gained by receiving E-PLAYS using an intention-to-treat approach. Costs and outcome data for the economic analysis will be collected prospectively during the trial using proxy-reported questionnaires at baseline and at each follow-up.

The primary analysis will be conducted using the CHU-9D [30, 31] which is a paediatric generic preference-based measure of quality of life that includes specific dimensions on school and joining in with activities and allows for the calculation of QALYs [31]. To ensure comparability with similar interventions, a secondary analysis will be conducted using the EQ-5D-Y [32]. Both instruments will be collected from proxies at baseline and at each follow-up. Mean within-trial costs and benefits will be calculated using regression methods adjusting for baseline covariates as well as any correlation between costs and utility. Multiple imputation methods will be used to deal with missing data if appropriate. Uncertainty will be described using confidence intervals and cost effectiveness acceptability curves (CEACs). A range of sensitivity analyses will be conducted to test the robustness of the results under different scenarios.

The bespoke resource use questionnaire developed for the feasibility trial of EPLAYs will be used. Healthcare resource use will be presented for both arms in terms of mean value, standard deviation and mean difference (with 95% confidence interval) between the groups. The cost of the intervention will be estimated according to treatment and resource use costs. Treatment costs will include staff, equipment and software costs. Unit costs will be derived from established national costing sources such as NHS Reference Costs and PSSRU Unit costs of health and social care. Unit costs will be multiplied by resource use to obtain a total cost for each patient (pupil).

The cost of delivering E-PLAYs was estimated in the feasibility trial. To confirm this, a costing exercise will be undertaken taking a bottom-up approach to identify and place a value on the constituent parts of the intervention delivery, e.g., staff and training costs, to estimate total cost both in monetary terms and time required including that of existing school staff.

The results of the trial will provide an estimate of the relative effect of E-PLAYs compared with usual care for the time horizon of the trial. However, there is potential for the impact of the intervention to extend far beyond what is measurable during the trial period, for instance, long-term educational outcomes and future criminal activity/anti-social behaviour. We will consider existing models that link the shorter-term outcomes of the trial, for example behavioural problems as measured by the SDQ, to longer term outcomes. One potential such model is the Dartington model [40] which could be used as the basis for linking short term outcomes to longer term educational attainment, future criminal activity and labour market productivity, though there are possibly other models available. We will use any identified models to examine the likely additional costs and benefits of the intervention over the longer term. As with the within trial analysis, health and educational effects will be presented separately and the potential values of the outcomes will be explored for both sectors. A discount rate of 3.5% will be applied for costs and outcomes.

### Data management

#### Data collection, management and verification

The five primary sources of quantitative data for this trial are:

1. Data collected by research assistants during school visits at baseline, 15–20 weeks and 35–40 weeks. The TPS and ERRNI will be audio recorded, with these recording being subsequently scored, and the relevant data entered into the REDCap database. All of the other Research Assistant completed measures (CELF-5, Droodles and Communication Test) will be entered into the REDCap database after testing.
2. Data collected from the teachers of participating focal children at baseline, 15–20 weeks and 35–40 weeks (CCC-2 and SDQ). These data will be collected via online Qualtrics surveys sent directly to teachers of participating focal children.
3. Data collected from the parents of participating focal children at baseline, 15–20 weeks and 35–40 weeks. Parent/household demographic data (e.g. parent/carer employment, parent/carer ethnicity etc.) are collected as part of the paper consent forms completed by parents of participating focal children. These forms are returned to the research team who enter these into the REDCap database. Parent completed data for the economic evaluation (i.e. EQ-5D-Y, CHU-9D and resource use) will be collected electronically (via direct entry into the trial REDCap database), or via paper questionnaires (which are subsequently returned to the trial team and entered into the trial database)
4. Data collected directly from the schools of participating children at baseline (provided for both focal and partner children). These data (e.g. age, gender, ethnicity etc.) are entered into password protected spreadsheets by school staff (one for each school). These are then securely shared with the research team.
5. Data collected by research assistants during intervention fidelity assessments.

Monitoring data: E-PLAYS-2 software will record the content, duration and number of intervention sessions each child receives using a unique login ID. Access to the E-PLAYS information is password protected and will be accessed on University computers with Bitlocker Windows security. Data within the E-PLAYS software is anonymised.

Most of the quantitative trial data will be stored and managed using REDCap (Research Electronic Data Capture). REDCap is a secure, web-based software platform designed to support data capture for research studies, providing 1) an intuitive interface for validated data capture; 2) audit trails for tracking data manipulation and export procedures; 3) automated export procedures for seamless data downloads to common statistical packages; and 4) procedures for data integration and interoperability with external sources [41, 42]. Data provided by teachers (CCC-2 and SDQ responses at baseline, 15–20 weeks, and 35–40 weeks) will be collected using a bespoke Qualtrics questionnaire [43] created by the research team at YTU. Data provided by schools via password protected Excel spreadsheets will serve as the raw data for these variables.

Validation of the quantitative data will be implemented as part of the REDCap and Qualtrics systems, so that data will be checked at the point of data entry. The validation rules implemented as part of the REDCap system were reviewed and agreed by the trial statistician and health economist prior to the start of data entry. The trial statistician and health economist have permissions to download the data stored in REDCap, saving these exports to secure password protected servers managed by YTU. Electronic datasets from the Qualtrics survey will be accessed directly from the Qualtrics software by the trial statistician, with these again being saved locally to YTU managed servers. Electronic datasets (.xlsx format) completed by schools will be stored in a central location accessible by the research team at YTU (including the trial statistician and health economist).

All quantitative data (REDCap, Qualtrics and school completed data stored on YTU servers) will be imported into statistical software (precise details reported in any outputs/reports). Further checks to investigate the consistency and completeness of the data will be undertaken. Any anomalies identified during these processes will be documented and resolved in accordance with the procedures outlined in YTU SOP S02: Statistical Quality Control. Any changes to the analysis data will be detailed in an assumptions log as described in YTU SOP S02: Statistical Quality Control.

There will also be qualitative data from interviews, surveys and structured observations in the form of audio recordings. Recordings will be securely transferred to the transcription company via a secure file transfer service. Audio recordings will be deleted once anonymised transcriptions have been received by the research team.

### Access to Data

The final anonymised trial dataset will be available to all trial team members/investigators if a formal request describing their plans is approved by the Trial Management Group. To ensure confidentiality, data dispersed to trial team members will be blinded of any identifying participant information. Appropriate anonymised datasets will be made available in a public repository, such as the UK Data Archive. Any participants that do not have explicit consent in place for publicly sharing anonymised data will have their data removed from any publicly available datasets.

### Data protection

The University of York will be the Data Controller who also processes data. Data subjects are the participants in the evaluation, which includes children in participating schools, their parents/carers and staff members in participating schools. Personal data will be processed under Article 6 (1) (e) (*Processing necessary for the performance of a task carried out in the public interest*) and Special Category data under Article 9 (2) (j) (*Processing necessary for … scientific … research purposes)* of the General Data Protection Regulation (GDPR; 2018). Any sharing of data between research team institutions will be made explicit in all participant information sheets and will be based on the procedures given in relevant Data Sharing Agreements. The study consent form will include optional statements affirming agreement with sharing anonymised data.

Potential participants of the trial will be informed about the research via an information sheet sent on behalf of the research team by Schools to parents/carers/children/staff. Parents/carers willing for their child to participate will provide written informed consent. Paper consent forms will be securely transported and stored in a locked filing cabinet at the University of Bedfordshire. A unique trial identification number (Trial/Child ID) will be generated for each participant. Data sharing agreements will be put in place with participating Schools before data transfer.

Recordings comprising audio-recordings from focus groups and interviews will be removed/deleted from audio-recorders by research assistants and stored on an encrypted flash drive (memory stick) before being transferred to university laptops compliant with university security regulations. Recordings will be securely transferred to the transcription company via a secure file transfer service. Audio recordings will be deleted once anonymised transcriptions have been received.

The dataset for statistical analysis will hold pseudonymised data. No Schools, staff members, or children will be identifiable in the report or dissemination of any results. Electronic data and paper documents including identifiable personal child data will be securely archived and disposed of by the research team five years after the end of the study (2029). Identifiable personal data about adult data subjects (e.g., parents/carers, school staff) will be kept for five years after the end of the study (2029). Pseudonymised electronic data and paper documents will be kept indefinitely.

### Ethics and regulatory considerations

- Ethical approval for the trial has been received from University of Bedfordshire, institute for Health Research Ethics Committee.
- The proposed study will be conducted in accordance with ICH Good Clinical Practice guidelines.
- A Memorandum of Understanding signed by schools will cover the requirements of the trial.
- Data Sharing Agreements (DSAs) will be signed by the University of Bedfordshire and each participating school.

*Ethical amendments and reporting.*

Substantial amendments will require approval by both NIHR in the first instance, and where necessary the Institute for Health Research ethics committee. All correspondence with the ethics committee and NIHR will be retained in the Trial Master File (TMF). Any changes relevant to schools will be communicated in writing at the earliest opportunity following approval.

### Trial monitoring

#### Protocol amendments

Protocol amendments will be approved by the Chief Investigator, sponsor, Trial Steering Committee and funder and then submitted to the ethics committee (if necessary).

### Protocol compliance and breaches

Accidental protocol deviations will be documented on the relevant forms and reported to the CI.

### Trial management group

The Trial Management Group (TMG) will be the decision-making body who will be responsible for the day-to-day running and management of the trial. The TMG will comprise the Chief Investigator, the co-applicants, the trial manager and other key members of the research team. The Trial Management Group will meet at least monthly.

### Trial steering committee

A Trial Steering Committee (TSC) will be established to govern the conduct of this study. This committee will function in accordance with YTU SOPs. The TSC will be led by an independent chair, a senior academic with relevant expertise and will comprise 75% independent members (as per NIHR’s definition https://www.nihr.ac.uk/documents/research-governance-guidelines/12154). The TSC will meet approximately every 6 months from the start of the trial.

### Advisory group (Public and Patient Involvement)

An advisory group will input into the trial and advise on matters such as recruiting a diverse sample, producing an accessible Participant Information Sheet and other relevant participant-facing study documents, support for teaching assistants and dissemination of our findings to participants and the public. The advisory group will comprise a mix of parents of children with SCD, teachers, speech and language therapists and relevant charity representatives. All members of the advisory group will be supported by a dedicated research team member. They will plan activities such as the preparation of information sheets and newsletters and other promotion of E-PLAYS. The dedicated research team member will provide feedback on these activities and their impact and will plan to distribute and promote E-PLAYS nationally if it is found to be effective at the end of the study.

### Adverse events and safeguarding

#### Serious Adverse Events (SAEs) and Adverse Events (AEs)

Due to the nature of participant involvement no serious adverse events or adverse events that are unexpected and related are anticipated. However, the study team will monitor adverse events throughout the study.

### Expected Events

This is a low-risk study and the trial team has not identified any adverse events that could be related to the intervention, therefore this will be determined on a case by case basis by the Chief Investigator. It is expected that there may be unrelated incidents of hospitalisations, illnesses, disabling/incapacitating/life-threatening conditions, other common illnesses and rarely deaths in the study population.

### Related Events

An event is defined as ‘related’ if the event was due to the administration of any research procedure. The relatedness of an event will be reviewed by the Chief Investigator and the Trial Steering Committee. An ‘unexpected event’ is defined as a type of event not listed in the protocol as an expected occurrence.

### Reporting of adverse events

Details of any SAEs or AEs reported to the study team by the participants will be considered by the Chief Investigator and the trial team. All AEs/SAEs will be recorded and reported to the Sponsor immediately upon knowledge of the event or as soon as is practicably possible to do so, and the Trial Steering Group and Trial Management Group at the next scheduled meetings. Any SAE which is unexpected and related will be reported immediately upon knowledge of the event or as soon as is practicably possible to do so to the Sponsor and Trial Steering Committee and will be reported to the Research Ethics Committee within fifteen days of the unexpected and related SAE being reported.

### Child safeguarding issue

In the very rare circumstance where a child safeguarding issue is suspected, for example during data collection, a set procedure will be followed, including contacting Chief Investigator Dr Suzanne Murphy. The child’s school and parents/carers will then be informed accordingly. Both the school’s and the University of Bedfordshire’s usual safeguarding policy will then be followed. A SOP will be written to detail these arrangements.

### Dissemination policy

On completion of the trial, the data will be analysed and a Final Trial Report will be prepared for NIHR and submitted after ratification by the TSC. We will publish the trial results in peer-reviewed journals irrespective of the findings. NIHR will be acknowledged as the funders in all publications. Participants will be provided with a report of the findings written in a style accessible for lay people, which will be accessible via schools. We will also provide on-going reports through our website as the trial progresses.

In order to disseminate E-PLAYS to professionals, we will offer workshops with the Royal College of Speech and Language Therapists and the children’s communication charity Speech and Language UK: Changing Young Lives. We will also publicise through National Association of Professionals concerned with Language Impaired Children (NAPLIC), Autistica, the National Autistic Society and the Communication Trust Consortium. We will also apply to have E-PLAYS registered on websites listing and reviewing evidence-based language interventions e.g., Education Endowment Foundation, the Learning Foundation. Special Educational Needs and Disabilities (SEND) teams in local authorities and CCGs are likely to be responsive to efforts to distribute a cost-free product. Should E-PLAYS prove to be effective at the end of this trial, distribution and implementation could start at once as it is a web-based intervention.

## Discussion

The E-PLAYS-2 trial aims to definitively test the effectiveness and cost-effectiveness of the E-PLAYS programme for children with social communication difficulties (SCDs). Should E-PLAYS prove to be effective, it will be offered as one of few evidence-based interventions available to schools and speech and language therapists.

Educationalists have long advocated computerised approaches as having considerable advantages for children with SCD. In spite of this, technological approaches have rarely been used and are widely seen as a missed opportunity [19, 44]. There is a lack of interventions for children with social communication difficulties, and language therapies as a whole have attracted little research funding (Bishop, 2010). However, with the recent COVID pandemic, the importance of IT devices and internet connectivity to schools has taken centre-stage. There have been calls to provide schools with more and better IT equipment to which the Government has responded with a £1bn package [72]. This recent recognition of the importance of technology for schools together with increased training and interest of school staff means that we believe they are likely to be receptive to computerised interventions.

### Sample selection

Wieckowski and White [20] in their extensive review of technological interventions for children with social communication impairments, commented that the focus in this field has been overwhelmingly on children with autism spectrum disorder (ASD); very few studies have evaluated technology use for the broader group of children with social communication difficulties. Furthermore, reviews have reported that participants who are male, white, and from professional-class backgrounds tend to be over-represented in ASD studies [45] and that it is likely that those who are most socially disadvantaged access speech and language therapy (SLT) services the least [46].

The E-PLAYS trial will aim to recruit as wide a variety of children with social communication difficulties as possible and will not limit the sample to those with an ASD diagnosis and/or in receipt of SLT service support. Teachers select the children in their class that they believe would benefit from E-PLAYS using a short questionnaire; this selection process aims to replicate conditions in the real-world in which schools are unlikely to have the resources to undertake a detailed assessment.

### Study strengths and limitations

As far as we are aware, this is one of few major trials investigating an intervention targeting social communication difficulties in young children. A major strength of the study is that we will use a blinded outcome measure to assess children’s language pre- and post-test. Pragmatic language skills are difficult to assess as they manifest only during dynamic social interaction, therefore, testing with standardised questionnaires may not be appropriate [47]. In spite of this, much social communication literature is based on non-blinded parent-, teacher- or clinician-report [48]. To address this limitation, we are using measures administered by independent, blinded outcome assessors; the TPS and CELF-5 subscales administered by blinded research assistants. We will also collect parent and teacher reports for comparison with other studies.

Another strength of the study is that we will be able to obtain a precise measure of the number and timing of E-PLAYS sessions delivered for fidelity purposes as this will be automatically recorded by the software. This is preferable to alternative methods such as asking teaching assistants to record sessions or keep a diary. This reporting will be supplemented with live observations to paint a detailed picture of intervention delivery.

A limitation of the study is that it is impossible to blind participants, parents and teachers due to the nature of the intervention. However, the primary outcome measure, the TPS, is administered by blinded research assistants and the trial statisticians remain blinded to mitigate any impacts.

## Conclusion

Against a backdrop in 2020 and 2021 where children’s socialisation with peers, communication skills and peer relations have suffered and the most deprived individuals have been hit the hardest, E-PLAYS is particularly timely. Its aim is to develop children’s social and collaborative skills by making novel use of technology; we believe it is likely to be welcomed by schools, parents and children.

## Data Availability

No datasets were generated or analysed during the current study.

## Abbreviations

AE: Adverse event
CACE: Complier Average Causal Effect
CEAC: Cost-Effectiveness Acceptability Curve
CCC-2: Children’s Communication Checklist
CELF-5: Clinical Evaluation of Language Fundamentals-5
CHU-9D: Child Health Utility questionnaire
CI: Chief Investigator
CONSORT: Consolidated Standards of Reporting Trials
CRF: Case Report Form
DSA: Data Sharing Agreement
EAL: English as an Additional Language
ECHP: Education, Health and Care Plan (outlining special educational needs)
ERRNI: Expression, Reception and Recall of Narrative Instrument
E-PLAYS-2: Enhancing Pragmatic Language skills for Young children with Social communication difficulties (2)
EQ-5D-Y: European Quality of Life-5 Dimension
FSM: Free School Meals
GCP: Good Clinical Practice
GDPR: General Data Protection Regulation
ISRCTN: International Standard Randomised Controlled Trials Number
ICC: Intra-Cluster Correlation
ITT: Intention To Treat
MAT: Multi-academy trust
MRC: Medical Research Council
MOU: Memorandum of Understanding
NHS: National Health Service
NIHR: National Institute for Health Research
PIS: Participant Information Sheet
PSSR: Personal Social Services Research Unit
QALY: Quality-Adjusted Life Years
RA: Research Assistant
RCT: Randomised Control Trial
SAE: Serious Adverse event
SCD: Social communication difficulties
SD: Standard Deviation
SEND: Special Educational Needs and Disability
SENCO: Special Educational Needs Co-ordinator
SDQ: Strengths and Difficulties Questionnaire
SOP: Standard Operating Procedure
TA: Teaching Assistant
TMF: Trial Master File
TMG: Trial Management Group
TPS: Test of Pragmatic Skills
TSC: Trial Steering Committee
UK: United Kingdom
YTU: York Trials Unit

## References

[1] Hwa-Froelich DA (2015). Social Communication Development and Disorders.

[2] Skuse DH, Mandy W, Steer C, Miller LL, Goodman R, Lawrence K, Emond A, Golding J (2009). Social communication competence and functional adaptation in a general population of children. J Am Acad Child Adolesc Psychiatry.

[3] Laws G, Bates G, Feuerstein M, Mason-Apps E, White C (2012). Peer acceptance of children with language and communication impairments in a mainstream primary school: Associations with type of language difficulty, problem behaviours and a change in placement organization. Child Lang Teach Ther.

[4] Mok PLH, Pickles A, Durkin K, Conti-Ramsden G (2014). Longitudinal trajectories of peer relations in children with specific language impairment. J Child Psychol Psychiatry.

[5] Ketelaars MP, Cuperus J, Jansonius K, Verhoeven L (2010). Pragmatic language impairment and associated behavioural problems. Int J Lang Commun Disord Disorders.

[6] Donno R, Parker G, Gilmour J, Skuse D (2010). Social communication deficits in disruptive primary-school children. Brit J Psychiatry.

[7] Gilmour J, Hill B, Place M, Skuse D (2004). Social communication deficits in conductdisorder: a clinical and community survey. J Child Psychol Psychiatry..

[8] Brinton B, Fujiki M, Montague EC, Hanton JL (2000). Children with language impairment in cooperative work groups: A pilot study. Speech Hear Serv Sch.

[9] Murphy SM, Faulkner DM, Farley LR (2014). The behaviour of young children with social communication disorders during dyadic interaction with peers. J Abnorm Child Psychol.

[10] Whitehouse AJO, Watt HJ, Line EA, Bishop DVM (2009). Adult psychosocial outcomes of children with specific language impairment, pragmatic language impairment and autism. Int J Lang Commun Disord.

[11] Sciberras E, Westrupp EM, Wake M, Nicholson JM, Lucas N, Mensah F (2015). Healthcare costs associated with language difficulties up to 9 years of age: Australian population-based study. Int J Speech Language Pathology.

[12] Kelly B, Williams S, Collins S, Mushtaq F, Mon-Williams M, Wright B, Mason D, Wright D (2017). The association between socioeconomic status and autism diagnosis in the United Kingdom for children aged 5–8 years of age: Findings from the Born in Bradford cohort. Autism..

[13] St CM, Goh FCM, Kok YS, Gibson JL (2019). Early Risk Factors and Emotional Difficulties in Children at Risk of Developmental Language Disorder: A Population Cohort Study. J Speech Lang.

[14] Law J, Rush R, McBean K (2014). The relative roles played by structural and pragmatic language skills in relation to behaviour in a population of primary school children from socially disadvantaged backgrounds. Emot Behav Diffic.

[15] Dockrell J, Lindsay G, Roulstone S, Law J (2014). Supporting children with speech, language and communication needs: an overview of the results of the Better communication Research Programme. J Lang Commun Disord Disorders.

[16] Lindsay G, Dockrell J, Law J. Roulstone (2011). Better communication research programme: 2nd interim report. Research Report DFE-RR172.

[17] Murphy S, Joffe V, Donald L, Radley J, Sunthararajah S, Welch C, Bell K, Messer D, Crafter S, Fairhurst C, Corbacho B, Rodgers S, Torgerson D (2021). Evaluating ‘Enhancing Pragmatic Language skills for Young children with Social communication impairments’ (E-PLAYS): a feasibility cluster-randomised controlled trial. Pilot Feasibility Study.

[18] Holt S, Yuill N (2014). Facilitating other-awareness in low-functioning children with autism and typically-developing pre-schoolers using dual-control technology. J Autism Dev Disord.

[19] Ploog BO, Scharf A, Nelson D, Brooks PJ (2013). Use of computer-assisted technologies (CAT) to enhance social, communicative, and language development in children with autism spectrum disorders. J Autism Dev Disord.

[20] Wieckowski A, White S (2017). Application of technology to social communication impairment in childhood and adolescence. Neurosci Biobehav Rev.

[21] Howe C (2010). Peer groups and children’s development.

[22] Kimhi Y, Bauminger-Zviely N (2012). Collaborative problem solving in young typical development and HFASD. J Autism Dev Disord.

[23] Murphy SM, Faulkner DM, Reynolds LR (2014). A randomised controlled trial of a computerised intervention for children with social communication difficulties to support peer collaboration. Res Dev Disabilities.

[24] Bishop DVM. Expression, Reception and Recall of Narrative Instrument (ERRNI): Pearson; 2004.

[25] Saghaei M, Saghaei S (2011). Implementation of an open-source customizable minimization program for allocation of patients to parallel groups in clinical trials. J Biomed Sci Eng.

[26] Shulman BB (1986). Test of Pragmatic Skills-Revised.

[27] Wiig EH, Semel E, Secord WA (2013). Clinical Evaluation of Language Fundamentals®-Fifth Edition (CELF®-5).

[28] Carmiol A, Vinden P (2013). Enhancing preschoolers’ understanding of ambiguity in communication: a training study on misunderstandings. Merrill-Palmer Q.

[29] Miller SA, Hardin CA, Montgomery DE (2003). Young children’s understanding of the conditions for knowledge acquisition. J Cogn Dev.

[30] Stevens K (2009). Developing a descriptive system for a new preference-based measure of health-related quality of life for children. Qual Life Res.

[31] Stevens K (2011). Assessing performance of a new generic measure of health-related quality of life for children and refining it for use in health state valuation. Appl Health Econ..

[32] Wille N, Badia X, Bonsel G, Burström K, Cavrini G, Devlin N, Egmar AC, Greiner W, Gusi N, Herdman M, Jelsma J (2010). Development of the EQ-5D-Y: a child-friendly version of the EQ-5D. Qual Life Res..

[33] Bishop DVM (2003). The Children’s Communication Checklist.

[34] Goodman R (2001). Psychometric properties of Strengths and Difficulties Questionnaire. J Am Acad Child Adolesc Psychiatry.

[35] StataCorp (2023). Stata Statistical Software: Release 18.

[36] Liu Q, Shepherd BE, Li C, Harrell FE (2017). Modelling continuous response variables using ordinal regression. Stat Med.

[37] Moore G, Audrey S, Barker M, Bond L, Bonell C, Hardeman W, Moore L, O’Cathain A, Tinati T, Wight D, Baird J (2014). Process evaluation of complex interventions UK Medical Research Council (MRC) guidance.

[38] Yin RK (2018). Case study research and applications: Design and methods.

[39] Braun V, Clarke V (2019). Reflecting on reflexive thematic analysis. Qual Res Sport Exerc Health.

[40] Little, M. et al T. Investing in Children: An Overview. Social Research Unit: Dartington (UK). www.wsipp.wa.gov/TechnicalDocumentation/Overview of WSIPPs Benefit-Cost Model.pdf Accessed 6 Feb 2024

[41] Harris PA, Taylor R, Thielke R, Payne J, Gonzalez N, Conde JG (2009). Research electronic data capture (REDCap) – A metadata-driven methodology and workflow process for providing translational research informatics support. J Biomed Inform.

[42] Harris PA, Taylor R, Minor BL, Elliott V, Fernandez M (2019). The REDCap consortium: Building an international community of software partners. J Biomed Inform.

[43] Qualtrics, Provo, UT, USA. https://www.qualtrics.com

[44] Piper AM, O'Brien E, Morris MR, Winograd T. SIDES: a cooperative tabletop computer game for social skills development. The 20th anniversary conference on Computer supported cooperative work, 4–8 November Banff, Alberta, Canada: ACM; 2006; 1–10.

[45] Safer- Lichtenstein J, Hamilton JC, McIntyre LL (2019). (2019) Examining Demographics in Randomized Controlled Trials of Group-Based Social Skills Interventions for Individuals with ASD. J Autism Dev Disor.

[46] Law J, Reilly S, Snow P (2013). Child Speech, Language and Communication need re-examined in a public health context. Int J Lang Commun Disord.

[47] Norbury CF (2014). Practitioner review: Social (pragmatic) communication disorderconceptualization, evidence and clinical implications. J Child Psychol Psychiatry..

[48] McConachie H, Parr JR, Glod M, Hanratty J, Livingstone N, Oono IP, Robalino S, Baird G, Beresford B, Charman T, Garland D, Green J, Gringras P, Jones G, Law J, Le Couteur AS, Macdonald G, McColl EM, Morris C, Rodgers J, Simonoff E, Terwee CB, Williams K (2015). Systematic review of tools to measure outcomes for young children with autism spectrum disorder. Health Technology Assessment..

